# HbA1c shows good correlation with regular post-prandial pre-exercise blood glucose measures in active individuals with type 2 diabetes mellitus

**DOI:** 10.1186/1758-5996-7-S1-A65

**Published:** 2015-11-11

**Authors:** Guilherme Falcão Mendes, Tulio Cesar Lins, Jane Dullius

**Affiliations:** 1 Universidade de Brasília, Brasília, Brazil

## Aims

This study aimed to verify the correlation between annual average glycated hemoglobin (HbA1c) and mean capillary blood glucose (BG) with post-prandial and post-exercise in active adults with type 2 diabetes mellitus (T2D) participants of a diabetes education program with emphasis on supervised exercise.

## Methods

The data was collected in a cross-sectional study with 103 T2D adults during the year 2009 (April to November). The Program had provided two weekly sessions of multidisciplinary health educational interventions with several modalities of supervised physical exercise (60min each one) and register of post-prandial (i.e. pre-exercise) and post-exercise BG. HbA1c between the stage of the study: beginning (I), 1 month recess ‘washout effect’ (II) and closure of the annual activities (III); mean post-prandial and post-exercise BG were calculated for these respective stages. Continuous variables were expressed as mean and standard deviation and analyzed by multivariate regression test (p < 0.05 assumed).

## Results

The annual HbA1c average was 7.5±1.5% with 42.7% of individuals <7.0%. The average post-prandial BG at the stage 1 was 161.25±56.65 mg/dL and at the stage III was 157.79±49.42 mg/dL (p>0.05), while the stage 1 average post-exercise BG was 132.45±42.47 mg/dL and at the stage III was 125.50±40.11 mg/dL, a significant difference (p=0.022). Finally, when multivariate linear regression was performed between HbA1c and the mean BGs throughout the year, the HbA1c value had modest and significant predictor (R^2^=0.59, p<0.001), in which the mean post-exercise BGs differed more from HbA1c (t=-2.07, p=0.041). That is, post-prandial BG before the exercise session better represents the average blood glucose estimated by HbA1c, and the exercises promote reductions that disperse from routine blood glucose levels, highlighting the importance of regular exercise to achieve a better control of diabetes.

## Conclusion

The average annual HbA1c relates much more to the average post-prandial (pre-exercise) BG than after the physical exercises, showing that the glucose levels obtained after intervention with supervised exercise are below the estimated average glucose offered by HbA1c and probably could reduce more the HbA1c if the exercise be practiced regularly.

